# Evaluation of Changes in Individual Community-Related Empowerment in Community Health Promotion Interventions in Estonia

**DOI:** 10.3390/ijerph8061772

**Published:** 2011-05-25

**Authors:** Anu Kasmel, Pernille Tanggaard

**Affiliations:** 1 Institute of Political Science and Governance, University of Tallinn, Narva mnt. 10-120, Tallinn, Estonia; 2 Institute of Public Health, University of Southern Denmark, Niels Bohrs Vej 9-10, 6700 Esbjerg, Denmark; E-Mail: ptandersen@health.sdu.dk

**Keywords:** individual community related empowerment, social change, health promotion, empowerment evaluation, Estonia, Eastern Europe

## Abstract

This study assessed changes in community members’ ratings of the dimensions of individual community related empowerment (ICRE) before and two years after the implementation of an empowerment expansion framework in three community health promotion initiatives within the Estonian context. We employed a self-administered questionnaire, the adapted *mobilisation scale–individual*. As the first step, we investigated the multidimensional nature of the ICRE construct and explored the validity and reliability (internal consistency) of the ICRE scale. Two datasets were used. The first dataset comprised a cross-sectional random sample of 1,000 inhabitants of Rapla County selected in 2003 from the National Population Register, which was used to confirm the composition of the dimensions of the scale and to examine the reliability of the dimensions. The second dataset comprised two waves of data: 120 participants from three health promotion programs in 2003 (pre-test) and 115 participants in 2005 (post-test), and the dataset was used to compare participants’ pre-test and post-test ratings of their levels of empowerment. The content validity ratio, determined using Lawshe’s formula, was high (0.98). Five dimensions of ICRE, *self-efficacy*, *intention*, *participation*, *motivation* and *critical awareness*, emerged from the factor analysis. The internal consistency (α) of the total empowerment scale was 0.86 (subscales *self-efficacy* α = 0.88, *intention* α = 0.83, *participation* α = 0.81 and *motivation* α = 0.69; *critical awareness* comprised only one item). The levels of ICRE dimensions measured after the application of the empowerment expansion framework were significantly more favourable for the dimensions *self-efficacy*, *participation*, *intention* and *motivation* to participate. We conclude that for Rapla community workgroups and networks, their ICRE was rendered more favourable after the implementation of the empowerment expansion framework.

## 1. Introduction

Researchers, practitioners and politicians have all recognised that empowerment represents a core concept in health promotion. Its importance has been highlighted in the Alma-Ata Declaration [1] and the Ottawa Charter [2]. Empowerment is defined as a process whereby communities, organisations and/or individuals are enabled to assume power to act effectively to change their lives [3–6]. Embedded within this definition is a point that is crucial for research on empowerment: the level of analysis at which the construct is conceptualised, *i.e.*, individual, organisational and/or community levels of empowerment [7]. The scope of the current study is at the level of individual communityrelated empowerment (ICRE).

Individual empowerment (IE) is the expression of the empowerment construct at the level of the person and reflects one’s freedom to decide what goals to pursue [8]. IE has been seen as an effective strategy in improving employees’ health [9], empowering low income mothers [10], enabling cancer patients [11], empowering young people [12] and in improving adult outpatient mental health [13].

However, despite the impressive body of published studies on empowerment [3], health promotion practitioners still have few tools for the measurement of the empowerment construct. Many authors have argued that ICRE is a prerequisite for community empowerment and social change [8,14,15]. Therefore, in evaluating the empowering processes, the evaluation of community empowerment might be supported by and benefit from the measurement of ICRE.

The current study is a part of a wider evaluation that included two parallel assessments: an internal evaluation of the organizational domains of community empowerment and an external evaluation of the ICRE. To the best of our knowledge, no research on ICRE has previously been undertaken in Estonia.

### 1.1. Aim of the Study

The main aim of the current study was to assess changes in ICRE in a sample of community members two years after the application of the empowerment expansion framework (see below) in the community of Rapla, Estonia. The specific objectives were as follows:

1. to assess the construct validity of the ICRE scale;
2. to investigate the multidimensional nature of the ICRE construct;
3. to assess the reliability of the ICRE scale; and;
4. to assess changes in participants’ ratings of the dimensions of the ICRE before and after the application of the empowerment expansion framework.

### 1.2. Theoretical Framework

Several authors have defined empowerment as a construct that links individual strengths and competencies, natural helping systems, and proactive behaviours to matters of social policy and social change. Psychological empowerment is a process by which individuals gain control over their lives [4]. Empowerment is associated with feelings of competence to change a situation and with expectations of positive outcomes for one’s efforts [7,14,15]. Individual empowerment begins with an individual belief that what one is trying to accomplish is possible to achieve.

Researchers have noted that the development of a universal measure of individual empowerment may not be a feasible or appropriate goal, as empowerment differs among individuals, contexts and times [16]. Furthermore, empowerment is not understood as merely an individualistic characteristic; rather, it is related to perceived goals in one’s environment. The measurement of individual empowerment is problematic because empowerment may manifest in different forms of perceptions, behaviour, competencies and actions, and moreover, it may fluctuate over time [3].

Several researchers have identified somewhat different scales for the measurement of individual empowerment depending on the context and/or specificity of study group. Zimmerman [16] defined three components of IE: intrapersonal, interactional and behavioural. The intrapersonal component includes community-specific self-efficacy, motivation and intention to take action and control in an individual’s community. The interactional component refers to critical awareness and understanding of a given context. The behavioural component includes participation in collective action. In the context of ethnic identity, Gutierrez [17] specified group identification as a psychological component of empowerment for individuals. Parsons [18] identified characteristics of empowerment in the context of mental health services. One characteristic identified was the degree to which clients develop a critical awareness or critical thinking regarding system dynamics within the family or community with relation to power. McWhirter [19] described skill development, a characteristic that stresses skills in decision making and socialisation. Akey *et al*. [20] utilised data from 293 parents of children with disabilities living in three states in the USA and participating in family support programs aimed at empowering parents. The scale developed by these authors originally contained three subscales: attitudes related to control and competence, critical skills and knowledge, and participatory behaviour. Speer and Peterson [21] elaborated a 27-item scale for the measurement of IE and reported psychometric properties of a scale from a sample of 974 randomly selected people. They identified cognitive, emotional, and behavioural dimensions in community-organising contexts. The applicability of their measure was broad. However, they all recognised that modifications need to be made on the basis of the variety of contexts and settings in which empowerment may be applied.

Empowerment theory proposes that empowerment takes on different meanings in different settings [16]. The definition of ICRE employed in the current study combines multiple components: *self-efficacy* with self-confidence [22–24]; involvement in collective action (*participation*); *motivation* to be involved in community action [25]; willingness and *intention* to take action in the public domain [25]; and *critical awareness* that community issues are serious [9,26].

S*elf-efficacy* is an individual’s confidence in their personal capability to organise and execute the course of action required to deal with prospective situations and belief in their capability to regulate their motivation, thought processes, emotional stages and the social environment, as well as behavioural attainment [4,14,25]. It is the belief that one has the skills and ability to achieve goals accompanied by perceived improvements in knowledge and skills through participation in community problem-solving processes [27]. Perceived self-efficacy with regard to dealing with community issues is associated with a sense of community [28,29] and with social action on community issues [14,15,25]. Perceiving that one can solve community problems is a prerequisite for community involvement [30].

*Participation* is the involvement in any community action that an individual attends without pay to achieve a common goal and/or social change [25,31].

*Motivation* is the belief that one should participate in community problem-solving processes as a responsibility to others [25,32]. Thus, people are motivated by a sense of moral responsibility to redress practices or change conditions that they perceive to be unfair [33].

*Intention* to participate is an anticipated outcome that is intended or that guides one’s planned action [25].

*Critical awareness* is the sense of the importance of community issues and understanding of the purposes of community action [27]. Critical comprehension and knowledge of social and political contexts is a prerequisite for the cultivation of both individual and collective resources and skills related to social action [23].

Essentially, ICRE is an active type of community orientation in which an individual wishes and feels able to shape his/her role and context [16]. The applicability of this measure is broad, but modifications need to be made on the basis of the variety of social work settings in which empowerment may be applied.

### 1.3. Context and Settings

The current study was undertaken in Rapla County, Estonia, which is a small agrarian inland community of 37,000 inhabitants. There is high employment rate and relative poverty of Rapla County’s population is high in comparison to other regions of Estonia [34]. Several health promotion interventions have been initiated in Rapla, which have been directed to different health issues. Until the current study, previous assessments of health promotion initiatives were mainly focused on measuring changes in health outcomes. In 2002 an empowerment expansion study was designed simultaneously to expand empowerment in communities and to assess changes in the organizational domains of community empowerment and also in ICRE of the participants involved in community health promotion programs [35]. This evaluation was premised on a two-pronged approach that comprised an internal evaluation undertaken by the community members and an external evaluation undertaken by a person external to the programs. The current study is an external evaluation of the ICRE. The study involved participants from three community health promotion initiatives in Rapla County: the *Safe Community* program; *Drug Abuse and AIDS Prevention* programme; and *Elderly Quality of Life* program. Short description of the essence of community health promotion interventions is presented in Table 1.

### 1.4. Empowerment Expansion Framework

**Figure 1.**
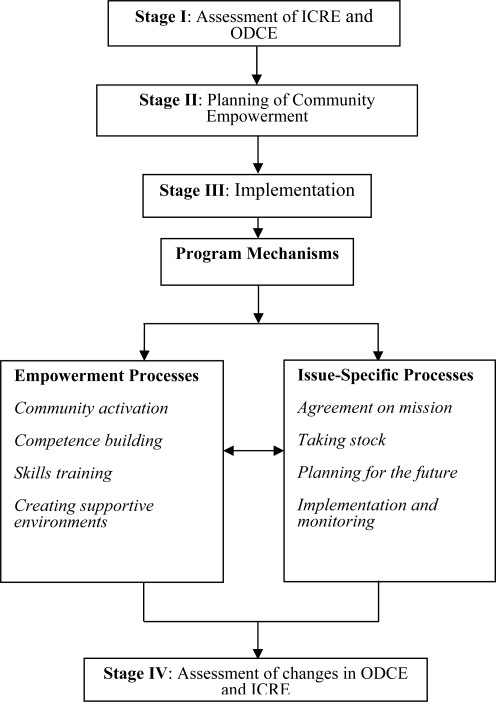
Empowerment expansion framework* [35].

The community workgroups constructed an empowerment expansion framework (Figure 1) to achieve and assess changes in empowerment and health in the three different programs that were being implemented [35]. The framework was based on models of empowerment evaluation, as suggested by Fettermann [36], and the *parallel tracks* model elaborated by Laverack [37]. Empowerment evaluation is a process through which community members in collaboration with health promotion practitioners learn to evaluate their own programs and work together toward the improvement of the quality of their common program using several pragmatic steps.

Empowerment evaluation is the use of evaluation concepts, techniques, and findings to foster improvement and self-determination [36]. The advantage of the model is that it suggests a clear step-by-step empowering guide. Its limitation is that it does not suggest how to measure changes in empowerment [35].

The ‘parallel tracks’ program planning model elaborated by Laverack [37] integrates an empowerment approach within an issue-specific approach ensuring focus on both empowerment process goals and issue-specific goals. The advantage of this model is that by clarifying and distinguishing empowerment domains, participants are able to easily assess changes in empowerment during an intervention course and measure empowerment domains. The limitation of this model is that it does not clearly demonstrate the precise steps involved in the empowerment approach.

The empowerment expansion framework creates an opportunity to simultaneously expand empowerment in a community, achieve expected outcomes related to community needs and evaluate changes in both tracks. The framework of the empowerment expansion comprised four stages:

Stage I—assessment of ICRE (undertaken by an external evaluator among the participants in the three community programs) and evaluation of the organisational domains of community empowerment (ODCE). The latter process is beyond the scope of the current study and is not reported here.

Stage II—planning of community empowerment. This included the formulation and statement of the empowerment expansion (undertaken by workgroups in each of the three community programs, where goals and objectives for the empowerment expansion were defined; measurable indicators and measurement processes were identified; and action plans were agreed upon).

Stage III—consisted of two parallel implementation processes:

- Empowerment expansion processes: included numerous activities targeted at the development of the four ODCE domains (Table 2). These processes were debated, formulated and planned by the community while being supported, facilitated and mediated by a health promotion practitioner and an internal evaluator; and
- Issue-specific processes: during which the guidelines for empowerment evaluation [36] were used, and four actions were undertaken:
  - agreement on an issue-specific mission;
  - taking stock (activities undertaken thus far were assessed, listed, analysed and rated, and an evaluation matrix was developed);
  - future planning (development of issue-specific goals and expected outcomes and formulation of an action plan). This also included the selection of measurement tools, indicators and a time-schedule for the issue-specific evaluation; *i.e.*, the creation of a system of processes and outcome monitoring;
  - implementation. Table 3 depicts some activities that were undertaken by community workgroups during the issue-specific processes.

Stage IV—evaluation of changes in community members’ ICRE (and assessment of the ODCE, which is not in the scope of the current paper).

## 2. Methods

### 2.1. Data

Two sets of data were collected. Ethical committee approval was not sought because in Estonia, studies that involve the voluntary participation of adults and have informed consent are exempt from further ethical approval.

The first dataset was used to investigate the multidimensional nature of the ICRE construct in the Estonian context and to assess the content validity and reliability of its dimensions. Questionnaires to be self-completed were mailed by regular post during April–May 2003 to a cross-sectional random sample of 1,000 inhabitants from Rapla County (selected from the National Population Register). Two reminders were subsequently mailed to those individuals who did not respond. The response rate was 67.1%. Respondents’ (n = 671) ages ranged from 17 to 71 years (Mean = 42; SD = 14.18). 392 (58.42%) female and 279 (41.58%) male respondents were included.

The second dataset was employed to assess changes in participants’ ratings of the dimensions of the ICRE. This sample consisted of all 120 voluntary participants from the three community programs who were involved in at least two program activities during the first intervention year of any of the three programs before the implementation of the empowerment expansion model. Two waves of the same self-administered questionnaire that was utilised for the first dataset were sent electronically: the first wave was sent one month before the first workshop related to application of the empowerment expansion framework in each community program separately (pre-test, 2003); and the second wave was sent after the last (third) workshop of the programs (post-test, January 2005). Additionally, two electronic reminders were sent to non-respondents, and phone interviews were undertaken with three individuals who did not respond electronically.

The pre-test was undertaken in 2003 (response rate 100%). Respondents’ (n = 120) ages ranged from 24 to 65 years (Mean = 43; SD = 10.9), and the sample comprised 78 (65%) women and 42 (35%) of men (Table 4). Of these participants, 22% had attained a primary level of education, 61% a secondary level, and 37% of the participants had a university education. With respect to the employment and affiliation of these individuals 19,16% were retired community members; 14,6% were people from the non-governmental sector; 10,83 worked in agriculture; 9,16% worked in the preschools and the same percentage in social work; 7,5% in the education system; 6,6% worked in the service and the same percentage in recreation sector; 3,33% were civil servants and students; 5% worked in the health care system; 2,50% were unemployed during the first measurement. In 2005, the post-test was undertaken. A total of 115 completed questionnaires were received during the post-test, which represented 95.8% of the pre-test participants. Five of the respondents who completed the pre-test had subsequently moved away from the community or were not available and, hence, were excluded from the current analysis. The mean age was 45 (SD = 10.51), and the sample consisted of 73 (63.48%) women and 42 (36.52%) of men.

### 2.2. Instruments: Questionnaire (Mobilization Scale–Individual)

There are few instruments that measure ICRE. For instance, Israel *et al.* [22] developed a 12-item *perceived control scale* to assess empowerment at individual, organisational and community levels (internal consistency α = 0.63). Similarly, Oman *et al.* [38] proposed a 6-item *community involvement scale*, and Reinigen *et al.* [12] suggested a *youth empowerment scale* (both with α = 0.78). Likewise, Spreitzer [9] developed a tool to evaluate IE in the workplace environment (12 items) that had a reliability coefficient 0.72. The present study utilised the *mobilization scale–individual* [39]. This scale was selected because most of the scale’s items emphasised participants’ perceptions of having the requisite abilities and motivations to make a difference in their communities. The original scale consists of nine subscales and 49 propositions. Five subscales (self-efficacy, participation, motivation, social assets and human capital) consisting 30 questions were selected as most appropriate for study context. The questionnaire was translated from English into Estonian language by two translators independently. Thereafter, the method of back-translation [40] was employed to determine the equivalence between the primary and secondary language tools. After the back-translation, the original and back-translated questionnaires were compared, and points of divergence were noted. The scale components were modified during a workshop in which Rapla community members were invited to respond to the items and discuss their cultural understanding and relevance to their community.

The content validity of the translated questionnaire was assessed by an expert panel of six health promotion experts. Each item in the questionnaire was discussed and rated as ‘essential’ (1) or ‘not necessary’ (0), and the content validity ratio (CVR) was calculated using the formula developed by Lawshe [41].

The final questionnaire consisted of 20 items rated on a Likert-type five-point scale (1 = ‘strongly agree’, the most favourable perception, to 5 = ‘strongly disagree’, the most unfavourable perception). The questionnaire considered the multidimensional nature of empowerment and allowed the assessment of the five dimensions of ICRE: *self-efficacy* related to an individual′s attitude toward social change in the community (7 items, e.g., “I have confidence in my capabilities to make the changes needed in my community”); *participation* in community activities (3 items, e.g., “I participate in community activities”); *intention* to become involved in community change (4 items, e.g., “I intend to take action in my community”); *motivation* to be involved (3 items, e.g., “I am motivated to get involved in my community”); and *critical awareness* that community issues are serious (one item, “I think that the problems in my community are serious”). Collectively, these dimensions provided a broad picture of ICRE.

### 2.3. Data Analysis

The software package SPSS 12.0 was used for the statistical analysis of the data. For the first objective of the study, to assess the construct validity of the ICRE scale, we employed Lawshe’s [41] formula: CVR = (n < item > e + n < item > e)/(N × n), where ne = number of experts rating *essential,* and N = number of items. To investigate the multidimensional nature of the ICRE construct within the Estonian context, the first dataset was used, for which factor analysis was employed to extract the factors by applying principal components analysis (varimax rotation) (Table 5). To assess the reliability of the ICRE scale, we used internal consistency coefficients measured by Cronbach’s alpha, which were undertaken twice: collectively for the total empowerment scale and individually for each of the five empowerment dimensions. To assess the changes in the participants’ ratings of the dimensions of the ICRE before and after the application of the empowerment expansion framework, we compared the pre-test and post-test results using an independent sample *t* test (one way ANOVA). Significance level was set at *p* < 0.5.

## 3. Results

### 3.1. Study Objective 1: Construct Validity of the ICRE Scale

Employing the formula suggested by Lawshe [41], the content validity ratio of the ICRE scale was 0.98, which is acceptable according to Davis [42] and Lawshe [41].

### 3.2. Study Objective 2: Dimensionality—the Multidimensional Nature of the ICRE

The dimensionality of the scale was evaluated by factor analysis (Table 5) using Kaiser’s criterion (eigenvalue > 1) [43]. The Kaiser-Meyer scale was .93, which is defined as very good [43]. Table 5 shows that five ICRE dimensions (factors) (*i.e.*, *self-efficacy*, *intention*, *participation*, *motivation* and *critical awareness*) emerged relatively clearly. However, two items (*intention*—“I pitch in when there is work to be done”, and *critical awareness*—“I feel that community issues are important”) exhibited fairy low loadings on their associated factors and relatively high loadings on other components. All remaining factors presented relatively strong loadings on their individual components. The factors consisted of three to seven items each (though only one item for the factor *critical awareness*). Collectively, the five dimensions explained 62.91% of the variance in ICRE. With respect to each of the individual dimensions, *self-efficacy* explained 20.37% of the variance, *intention* 16.96%, *participation* 11.97%, and *motivation* 7.22%. Their corresponding Eigen values (a common criterion for a dimension to be useful) were 8.52, 1.91, 1.25 and 1.02 [44].

### 3.3. Study Objective 3: Reliability of ICRE

Cronbach alpha coefficients were used to assess the reliability of the scale (Table 5). The internal consistency (Cronbach’s α) of the total scale was 0.859, which is very good [45]. Likewise, the internal consistencies of the three sub-scales comprising three dimensions (Self-efficacy 0.883; Intention 0.834; Participation 0.808) were very good. The Motivation dimension had a lower α (0.69), which is considered satisfactory. Critical awareness comprised only one item, and hence, α was not applicable.

### 3.4. Study Objective 4: Comparison of Pre- and Post-Test Ratings of ICRE Dimensions (Before and After the Application of the Empowerment Expansion Model)

Table 6 presents the means and standard deviations of the empowerment scale at the beginning of the application of the empowerment expansion framework and two years after its application. Generally, the means of the post-test (2005) for the five dimensions of empowerment and for total empowerment were more favourable than their pre-test means (2003). These changes were statistically significant for four of the five dimensions (*self-efficacy*, *participation*, *intention*, and *motivation*).

These findings suggested that respondents’ perceptions of their *self-efficacy* related to community problem-solving had increased in 2005 in comparison with the 2003 data. Participants’ confidence in their capabilities to undertake the required changes in their communities had increased, as respondents felt they could influence their communities to take action on important issues. Furthermore, respondents’ *motivation* to participate, *intention* to participate and actual *participation* in community activities had all increased. Similarly, respondents’ *critical awareness* of the seriousness of the community issues also exhibited a moderate increase, although the increase was not significant. Nevertheless, the 2003 data indicated that the *critical awareness* component of empowerment already presented a high value during the pre-test period, suggesting that for these three programs, respondents’ baseline awareness of community problem seriousness was already high before the initiation of the programs.

## 4. Discussion

Given that expansion of empowerment is frequently the primary objective of community health promotion programs, the positive change in ICRE among the participants of the programs investigated in this study is gratifying. The empowerment of communities has been recognised as a key purpose and critical function of health promotion initiatives [31,32,46,47]. Not surprisingly, numerous health promotion efforts have employed empowerment as a vehicle for their programs to combat a range of health and social problems. The types of such programs in which empowerment strategies have been used are diverse and are related to, e.g., violence against women [48], promotion of mental health [49], maternal and reproductive health among internally displaced communities [50], and the reduction of sex workers’ vulnerability to HIV/STDs [51]. Similarly, empowerment has been successfully utilised in decreasing the burden of lymphatic filariasis [52], in dengue prevention [53] and in the Healthy Cities movement [54].

However, few studies studies on this topic have previously been reported from eastern European countries, and there remains a gap in the empowerment literature in terms of research undertaken in (post-communist) countries that are in political and economic transition, such as Estonia. The current study bridges this gap, and while Estonia is classified economically as a high-income country [55], the current study was undertaken in a mainly rural area of Estonia where the relative poverty of the county’s population was high in comparison to other regions of Estonia [34]. In such settings, the empowerment of community members through program participation has been shown to be particularly important [56]. This is probably because ‘empowerment’ mechanisms engender a belief in oneself that is accompanied by taking responsibility in community development.

The current study employed an empowerment expansion framework based on models of empowerment evaluation and ‘parallel tracks’ program planning [36,37]. Using this framework, program participants from three community health promotion initiatives were empowered through: (1) a range of empowerment activities (e.g., community activation, competence building and skills training, in addition to the creation of supportive environments) and (2) a variety of issue-specific actions (e.g., agreement on a mission, taking stock, planning for the future, implementation and monitoring). The post-test findings indicated that the employed empowerment processes were associated with enhanced feelings regarding *self-efficacy* related to social change in the community, *participation* in community activities, *intention* to become involved in community change, *motivation* to be involved, and *critical awareness* that community issues are serious.

Tools for the measurement of ICRE are still under development. This study undertook an initial step towards examining ICRE in the Estonian context. Hence, we adopted a *mobilization scale–individual* [39] that measures individual community-related empowerment. We validated the scale, which demonstrated a satisfactory five-factor solution, confirming five specific dimensions of ICRE. Factor analysis indicated that the first factor, *self-efficacy,* was one of the strongest and most consistent. This is in agreement with findings reported in the USA by Rogers *et al.* [57], who studied the individual empowerment of 271 participants in self-help groups in six states. Our results are also in support of the findings of Wowra and McCarter [13], who validated an empowerment scale in an adult outpatient mental health population in South Carolina among 283 patients and similarly reported that self- efficacy was the strongest dimension.

Likewise, the dimensions *participation* and *intention* to participate in community actions imply the ability and willingness to participate, which is in agreement with the results of a study by Eklund [27] among two Finnish communities where she found increased *participation* and *intention* to participate in community initiatives after utilising empowerment strategies. The results of Bejerholm and Björkman’s research among people with mental illness entering supported employment in Sweden demonstrated a higher level of engagement in daily activities [58]. In a study undertaken by Röger *et al*. [59] among disadvantaged women in Germany, participation in community initiatives was found to be better among empowered individuals.

Our findings related to the dimension *motivation* to participate in the community, as an important component of ICRE, are in line with those of Mok *et al.* [11], who studied individual empowerment among Chinese cancer patients in Hong Kong. They found that the *motivation* dimension was critical for IE. Similarly, our findings regarding the dimension *critical awareness* are in agreement with the results of a study by Champeau and Shaw [60] in which they examined critical consciousness in the dynamics of a public health community collaboration around an HIV prevention media campaign for women in the USA and observed its importance in ICRE.

In our sample, the five factors described above emerged sufficiently clearly. Although the factor *critical awareness* comprised only one item, the ICRE scale proved to be valid and appropriate for the measurement of its dimensions. Furthermore, the reliability coefficient for the total empowerment scale (α = 0.859) demonstrated very good internal consistency [45].

Our findings confirmed that the framework for ICRE adapted and utilised in the current study is consistent with the definitions of ICRE offered by Zimmerman and Rappaport [25] and Bracht and Tsouros [31]. The features constituting ICRE included community members’ *self-efficacy* and confidence in their personal capability to organise and execute the course of action required to deal with community problems; *participation*, *motivation* and *intention* to participate without pay to achieve a common goal and/or social change in their community; and *critical awareness* as the sense of importance of community issues and understanding of the purpose of community action. Our findings are consistent with those of Bejerholm and Björkman [58], who studied people with mental illness entering supported employment and found that the use of multilevel empowerment approaches to health are required to support individual empowerment.

Comparison of the pre- and post-test scores for the five dimensions revealed that all dimensions were improved in the post-test. The findings demonstrated a significant increase both in total empowerment and in four (of the five) dimensions related to perceived ICRE. A somewhat unexpected finding was the absence of a significant change in the fifth dimension, *critical awareness* of community issues. A likely reason for this might be the relatively high score for *critical awareness* already prevalent in the pre-test, or alternatively, the provision of insufficient focus on this particular dimension in the intervention. Nevertheless, a slight increase was also observed in the dimension *critical awareness*. Thus, the ICRE scale was shown to allow the researchers and community members to determine the levels of ICRE as perceived by community members before and after the implementation of an empowerment expansion model in three community programs. Furthermore, it provided members of the community workgroups with valuable information about factors that could be modified to achieve even more favourable ICRE among participants in the community programs.

The current study has limitations. Respondents from three health promotion programs from one county in Estonia participated in the study. This geographical limitation means that similar assessments in other regions and with larger sample sizes would be required to confirm or refute the present findings. Additionally, the community workgroups and network members who participated in the current study consisted of individuals with a heightened sense of social responsibility and social activity, as suggested by the fact they were already involved in a range of community development processes. Further studies are needed to apply the framework in more and different communities. Similarly, individuals may be greatly influenced by the context and other unanticipated events in their communities, which might influence their ICRE. Although this study affirmed that ICRE became more favourable during the intervention period, it is not possible to conclude that this materialised due to the health promotion interventions that we assessed. Furthermore, due to the cross-sectional nature of the current study, the observed trends are associations and should not be viewed as causations.

This study holds important implications for health promotion practice. Health promotion practitioners working with community networks might benefit from scrutinising the ICRE dimensions in the specific community contexts in which they work in a precise manner. Furthermore, asserting community members’ empowerment status (and its dimensions) prior to a planned intervention could be beneficial for needs assessment exercises in terms of the dimensions of ICRE that might require strengthening to ensure effective program planning to meet the particular needs of the community members. Thus, the use of an empowerment expansion framework described here by communities could assist them in focussing on particular ICRE dimensions, which might then become essential and integral parts of a given community health promotion program. The findings of the present study suggested that to empower community members as part of a planned effort, community workgroups, together with local health promotion practitioners and evaluators, could direct their efforts to deliberately planned activities that would increase community members’ *self-efficacy*, *participation*, *motivation* and *intention* to participate in community actions, as well as *critical awareness* of community issues.

## 5. Conclusions

The current study aimed to assess changes in community members’ ratings of ICRE after two years of application of an empowerment expansion framework in Rapla County, Estonia. Comparison of the scores from pre- and post-tests revealed that all dimensions of ICRE were improved in the post-test. Our findings demonstrated a significant increase both in total empowerment and in four (of the five) dimensions of perceived ICRE, as well as a slight non-significant increase in one item, *critical awareness*. To clarify the concept of ICRE, the empowerment scale was adapted to the community context, and factor analysis was utilised to identify its dimensions. A five-dimension ICRE scale emerged from the factor analysis, which was congruent with results reported in the literature, confirming the usefulness of ICRE characterised by the context-specific dimensions of perceived *self-efficacy*, self-reported *participation*, *intention* and *motivation* to participate in community actions and *critical awareness* of the seriousness of community problems. The internal consistency of the ICRE scale was very good. However, future studies in different communities and community workgroups will be necessary to elaborate the scale as a measurement instrument for the assessment of ICRE in community empowerment initiatives. We conclude that for the investigated Rapla community workgroups and networks, ICRE was rendered more favourable after the implementation of the empowerment expansion framework among the three health promotion programs. However, the cross-sectional study design employed here does not allow the demonstration of a cause-and-effect relationship between the intervention and ICRE outcomes.

## Figures and Tables

**Table 1.** Study sample: three community health promotion and disease prevention initiatives in Estonia [35].

**Community Initiative**	**Description**
**Safe Community**	This program was initially a *bottom-up* initiative guided by a community workgroup. It later involved representatives from municipalities and decision makers from different sectors and had a large network in the county. It consisted of a combination of a *top-down* and *bottom-up* initiative financed on a yearly basis by a health promotion fund
**Drug Abuse and AIDS Prevention**	A *top-down* program initiated and planned nationally. It had national goals, objectives and a national action plan. This program was financed by the state budget and guided by a local coalition composed of representatives from different organisations, authorities and sectors in the county
**Elderly Quality of Life**	A *bottom-up* initiative. The workgroup consisted of elderly women who were interested in improving the life of elderly citizens in their community. The aim of the program was to avoid the exclusion of older people, and the group made efforts to keep elderly citizens involved socially

**Table 2.** Examples of empowerment expansion processes: ODCE domains and corresponding activities that were implemented [35].

**Domain**	**Activities**
**Community activation**	- Activities to support community members’ participation in community problem-solving processes - Involvement and engagement of more stakeholders - Motivation of new leaders - Creation and encouragement of new networks - Initiation and stimulation of new community groups among other processes
**Community competence**	- Training to improve the awareness and knowledge of community members to solve community problems - Distribution of information on good practices and evidence-based approaches - Information sharing to improve understanding of concepts, determinants and theories in health promotion among other processes
**Program management skills**	- Teaching program management and team building skills - Training for planning, implementation and evaluation techniques - Instruction on information use, dissemination and communication skills - Improving community groups, abilities and expertise in the use of evidence-based techniques in identifying, solving and managing their problems among other processes
**Creation of supportive environment**	- Training community members in lobbying skills - Advocating for political support and financial resources - Promoting better access to different foundations and expert resources - Improving participants’ abilities to maintain and sustain political changes and achieve a large amount of social support, among other processes

ODCE: organisational domains of community empowerment.

**Table 3.** Issue-specific processes: some activities undertaken by community workgroups [35].

**Community Initiative**	**Issue-Specific Activities**
**Safe Community**	- Organising safety campaigns - Teaching school-children traffic behaviour - Publishing printed materials for mothers of newborn babies on the prevention of babies’ injuries - Organising swimming courses to prevent drowning - Implementing safe school campaigns - Publishing printed materials for elderly persons to prevent falls - Distribution of grants to stimulate small prevention projects
**Drug Abuse and AIDS Prevention**	- Lobbying local policy makers to support the regulation of night sales of alcohol and to reduce youths’ access to alcohol - Organising alternative activities for youth (summer-camps, drug-free discos) - Implementing an anti-AIDS campaign and the distribution of condoms to young people - Producing printed material on sexual education for young people
**Elderly Quality of Life**	- Organising physical activity events in nature and in sport halls - Organising picnics and cultural outings - Inviting experts to talk on and debate health issues - Undertaking social support visits to peers - Implementing elderly Health Days

**Table 4.** Selected socio-demographic characteristics of respondents.

**Characteristics**	**Year 2003**		**Year 2005**	
	**N**	**%**	**N**	**%**
**Total**	120	100	115	95.8
**Gender**				
Male	42	35.00	42	36.52
Female	78	65.00	73	63.48
**Age**				
Range y	24–65		25–65	
Mean y SD	43 (10.90)		45 (10.51)	
**Education**				
Primary	22	18.33	19	16.52
Secondary	61	50.83	61	53.04
University	37	30.83	35	30.43
**Affiliation**				
Retired	23	19.16	21	18.26
Non-governmental sector	17	14.16	19	16.52
Agriculture sector	13	10.83	13	11.30
Pre-school	11	9.16	11	9.56
Social sector	11	9.16	11	9.56
Education sector	9	7.50	9	7.83
Recreation	8	6.66	8	6.96
Service	8	6.66	8	6.96
Students	7	5.83	5	4.35
Health care sector	6	5.00	6	5.22
Civil servants	4	3.33	4	3.48
Unemployed	3	2.50	-	-

**Table 5.** Assessment of the ICRE scale: principal components analysis.

**Dimension**	**Items**	**Component**				
		**1**	**2**	**3**	**4**	**5**
**1. Self-efficacy**	- I have the knowledge and skills to influence the community	0.774				
α = 0.883	- I have the ability to impact my community in important ways	0.771				
	- I have confidence in my capabilities to make needed changes in my community	0.755				
	- I am able to affect the area in which I live	0.743				
	- I can influence community members to take actions on important issues	0.671				
	- I have the knowledge and skills to gather information relevant to my community	0.647				
	- I know I can make a differences in my community	0.561				

**2. Intention**	- I want to get involved in my community		0.814			
α = 0.834	- I am willing to get involved in my community		0.786			
	- I am going to get involved in my community		0.759			
	- I intend to take action in my community		0.603			

**3. Participation**	- I participate in community activities			0.697		
α = 0.808	- I am involved in my community			0.562		
	- I volunteer for community projects			0.512		

**4. Motivation**	- I think it is important for me to get involved in my community				0.558	
α = 0.69	- I feel that efforts to address community issues are worthwhile				0.522	
	- I am motivated to get involved in my community				0.508	

**5. Critical awareness**	- I think that the problems in my community are serious					0.707

**Excluded**	- I pitch in when there is work to be done	0.230	0.396	0.485	0.249	0.310
	- I feel that community issues are important	0.136	0.374	0.406	0.462	

**Explained variance of ICRE** (%)		20.37	16.96	110.97	70.22	60.39

Extraction method: principal components analysis; rotation method: varimax with Kaiser Normalization.

**Table 6.** Comparison of participants’ ratings of ICRE and its dimensions before and after the application of the empowerment expansion framework (pre- and post-test).

	**Pre-Test 2003 N = 120**		**Post-Test 2005 N = 115**		***t*-value**	**DF**	***p***

	Mean	SD	Mean	SD			
Empowerment (Total scale)	1.87	0.37	1.82	0.35	3.179	225	0.002 *
Self-efficacy	2.12	0.43	2.07	0.41	2.345	225	0.020 *
Participation	1.70	0.56	1.64	0.52	2.245	225	0.026 *
Intention	2.02	0.54	2.01	0.49	3.192	225	0.002 *
Motivation	1.83	0.56	1.73	0.64	2.173	225	0.031 *
Critical awareness	1.68	0.33	1.62	0.31	1.668	225	0.097

All items rated on a Likert-type five-point scale (1 = ‘strongly agree’—most favourable perception, 5 = ‘strongly disagree’—most unfavourable perception);

* significant.

## References

[1] World Health Organisation (1978). Declaration of Alma-Ata.

[2] World Health Organisation (1986). Ottawa Charter for Health Promotion.

[3] Wallerstein N (2006). What Is the Evidence of Effectiveness of Empowerment to Improve Health?.

[4] Rappaport J (1984). Studies in empowerment: Introduction to the issue. Prev. Human Serv.

[5] Minkler M, Minkler M (2005). Improving Health through Community Organization and Community Building: A Health Education Perspective. Community Organizing & Community Building for Health.

[6] Fawcett SB, Paine-Andrews A, Francisco VT, Schultz JA, Richter KP, Berkley-Patton J, Fisher JL, Lewis RK, Lopez CM, Russos S, Rootmann I, Goodstadt M, Hyndman B, McQueen DV, Potvin L, Springhet J, Ziglio E (2001). Evaluating Community Initiatives for Health and Development. Evaluation in Health Promotion: Principles and Perspectives.

[7] Prilleltenski I (1994). Empowerment in mainstream psychology: Legitimacy, obstacles, and possibilities. Can. J. Psychol.

[8] Speer PW, Hughey J (1995). Community organizing: An ecological route to empowerment and power. Am. J. Community Psychol.

[9] Spreitzer GM (1995). Psychological empowerment in the workplace: Dimensions, measurement and validation. Acad. Manag. J.

[10] Becker J, Kovach AC, Gronseth DL (2004). Individual empowerment: how community health workers operationalize self-determination, self-sufficiency, and decision-making abilities of low-income mothers. J. Community Psychol.

[11] Mok E, Martinson I, Wong TKS (2004). Individual empowerment among Chinese cancer patients in Hong Kong. Western J. Nurs. Res.

[12] Reinigen B, Evans AE, Griffin SF, Valois RF, Vincent ML, Para-Medina D, Taylor1 DJ, Zullig KJ (2003). Development of a youth survey to measure risk behaviours, attitudes and assets: Examining multiple influences. Health Educ. Res.

[13] Wowra SA, McCarter R (1999). Validation of the empowerment scale with an outpatient mental health population. Psych. Serv.

[14] Florin P, Wandersman A (1990). An introduction to citizen participation, voluntary organizations, and community development: insights for empowerment through research. Am. J. Community Psychol.

[15] Chavis D, Wandersman A (1990). Sense of community in the urban environment: A catalyst for participation and community development. Am. J. Community Psychol.

[16] Zimmerman MA, Rappaport J, Seidman E (2000). Empowerment Theory: Psychological, Organizational and Community Levels of Analysis. Handbook of Community Psychology.

[17] Gutierrez LM (1995). Understanding the empowerment process: Does consciousness make a difference?. Soc. Work Res.

[18] Parsons R, Hera W, Wells LM (1999). Assessing Helping Processes and Client Outcomes in Empowerment Practice: Amplifying Client Voice and Satisfying Funding Sources. Empowerment Practice in Social Work: Developing Richer Conceptual Foundations.

[19] McWhirter EH (1991). Empowerment in counselling. J. Couns. Dev.

[20] Akey TM, Marquis JG, Ross ME (2000). Validation of Scores on the Psychological Empowerment Scale: A Measure of Empowerment for Parents of Children with a Disability. Educ. Psychol. Meas.

[21] Speer PW, Peterson NA (2000). Psychometric properties of an empowerment scale: Testing cognitive, emotional and behavioural domains. Soc. Work Res.

[22] Israel BA, Checkoway B, Schulz A, Zimmerman M (1994). Health education and community empowerment: Conceptualizing and measuring perceptions of individual, organizational, and community control. Health Educ. Quart.

[23] Kieffer C, Rappaport J, Swift C, Hess R (1984). Citizen Empowerment: A Developmental Perspective. Studies in Empowerment: Steps Toward Understanding and Action.

[24] Zimmerman MA (1990). Taking aim on empowerment research: On the distinction between individual and psychological conceptions. Am. J. Community Psychol.

[25] Zimmermann MA, Rappaport J (1988). Citizen participation, perceived control, and psychological empowerment. Am. J. Community Psychol.

[26] Dimidriades Z, Kufidu S (2004). Individual, job, organizational and contextual correlates of employment empowerment: some Greek evidence. Electron. J. Bus. Ethics Org. Stud.

[27] Eklund L (1999). From Citizen Participation towards Community Empowerment: An Analysis on Health Promotion from Citizens Perspective.

[28] Davidson WB, Cotter PR (1989). Sense of community and political participation. J. Community Psychol.

[29] McMillan B, Florin P, Stevenson J, Kerman B, Mitchell RE (1995). Empowerment praxis in community coalitions. Am. J. Community Psychol.

[30] Hirsch EL (1990). Sacrifice for the course: Group processes, recruitment and commitment in a student social movement. Am. Sociol. Rev.

[31] Bracht N, Tsouros A (1990). Principles and strategies of effective community participation. Health Prom. Int.

[32] Wallerstein N (1992). Powerlessness, empowerment, and health: Implications for health promotion programs. Am. J. Health Prom.

[33] Horvath P (1999). The organization of social action. Can. J. Psychol.

[34] Rapla Maavalitsus (2002). Rapla County and Its People.

[35] Kasmel A, Andersen PT (2011). Measurement of community empowerment in three community programs in Rapla (Estonia). Int. J. Environ. Res. Public Health.

[36] Fetterman DM, Fetterman DM, Kaftarian SJ, Wandersman A (1996). Empowerment Evaluation: An Introduction to Theory and Practice. Empowerment Evaluation: Knowledge and Tools for Self-Assessment and Accountability.

[37] Laverack G (1999). Addressing the Contradiction between Discourse and Practice in Health Promotion.

[38] Oman RF, Vesely SK, McLeroy KR (2002). Reliability and validity of the Youth Asset Survey (YAS). J. Adol. Health.

[39] Jakes S, Shannon L (2002). Community Assets Survey.

[40] Lin YH, Chen CY, Chiu PK (2005). Cross-Cultural Research and Back-Translation. Sport J.

[41] Lawshe CH (1975). A quantitative approach to content validity. Person. Psychol.

[42] Davis L (1992). Instrument review: Getting the most from your panel of experts. Appl. Nurs. Res.

[43] DeVellis RF (2003). Factor analysis, scale development theory and application. Appl. Soc. Res. Method Ser.

[44] Morgan GA, Griego OV (1998). Easy Use and Interpretation of SPSS for Windows.

[45] Sekaran U (1992). Research Methods for Business: A Skill Building Approach.

[46] El Ansari W, Phillips CJ (2001). Empowering health care workers in Africa: Partnerships in health—beyond the rhetoric towards a model. Critic. Pub. Health.

[47] Ridde V (2007). Reducing social inequalities in health: Public health, community health or health promotion?. Promot. Educ.

[48] Kalaca S, Dundar P (2010). Violence against women: The perspective of academic women. BMC Pub. Health.

[49] Taylor J, Jones RM, O’Reilly P, Oldfield W, Blackburn A (2010). The Station Community Mental Health Centre Inc: Nurturing and Empowering. Rural Rem. Health.

[50] Mullany LC, Lee TJ, Yone L, Lee CI, Teela KC, Paw P, Shwe Oo EK, Maung C, Kuiper H, Masenior NF, Beyrer C (2010). Impact of community-based maternal health workers on coverage of essential maternal health interventions among internally displaced communities in Eastern Burma: the MOM project. PLoS Med.

[51] Swendeman D, Basu I, Das S, Jana S, Rotheram-Borus MJ (2009). Empowering sex workers in India to reduce vulnerability to HIV and sexually transmitted diseases. Soc. Sci. Med.

[52] Rajendran R, Sunish IP, Munirathinam A, Ashok Kumar V, Tyagi BK (2010). Role of community empowerment in the elimination of lymphatic filariasis in south India. Trop. Biomed.

[53] Sanchez L, Perez D, Cruz G, Castro M, Kourí G, Shkedy Z, Vanlerberghe V, van der Stuyft P (2009). Intersectoral coordination, community empowerment and dengue prevention: Six years of controlled interventions in Playa Municipality, Havana, Cuba. Trop. Med. Int. Health.

[54] Heritage Z, Dooris M (2009). Community participation and empowerment in Healthy Cities. Health Promot. Int.

[55] The World Bank (2010). Country and Lending Groups.

[56] Smith MA, Garbharran H, Edwards MJ, O’Hara-Murdock P (2004). Health promotion and disease prevention through sanitation education in South Africa Zulu and Xhosa women. J. Transcult. Nurs.

[57] Rogers ES, Chamberlin J, Ellison ML, Crean T (1997). A consumer constructed scale to measure empowerment among users of mental health services. Psych. Serv.

[58] Bejerholm U, Björkman T (2010). Empowerment in supported employment research and practice: Is it relevant. Int J Soc Psychiatry.

[59] Röger U, Rütten A, Frahsa A, Abu-Omar K, Morgan A (2010). Differences in individual empowerment outcomes of socially disadvantaged women: Effects of mode of participation and structural changes in a physical activity promotion program. Int J Public Health.

[60] Champeau DA, Shaw SM (2002). Power, empowerment, and critical consciousness: Lessons learned from an advisory panel for an HIV awareness media campaign for women. Women Health.

